# Experimental use of a cellulosic biopolymer as a new material for suburethral sling in the treatment of stress urinary incontinence

**DOI:** 10.1590/S1677-5538.IBJU.2014.0155

**Published:** 2015

**Authors:** Roberto G. Lucena, Salvador V. C. Lima, José L. de A. Aguiar, Rogerson T. Andrade, Flávia C. M. Pinto, Fabio O. Vilar

**Affiliations:** 1 Núcleo de Cirurgia Experimental do Departamento de Cirurgia da Universidade Federal de Pernambuco, Recife, Pernambuco, Brasil

**Keywords:** Suburethral Slings, Urinary Incontinence, Cellulose, Biopolymers

## Abstract

**Purpose:**

To analyze the interaction between the cellulose exopolysaccharide (CEC) and urethral tissue when used as a pubovaginal sling.

**Materials and Methods:**

Forty Wistar rats were divided into four groups. In groups A and B the cellulose exopolysaccharide (CEC) was implanted around the urethral tissue (bladder neck below the upper margin) and the rats were sacrificed at 30 and 90 days. Similar procedure was used in groups C and D using a polypropylene mesh. After sacrifice bladder and urethra were sent for histological analysis. The histological parameters (inflammatory reaction) by evaluated by quantitative analysis. For collagen deposition analysis it was used stereological method.

**Results:**

The cellulose exopolysaccharide (CEC) was inert and well preserved at the implanted region at the time of examination. Morphologic alterations were not found at the CEC implant but some reactions of foreign body type were observed at the adjacent structures. In some areas a process of neovascular formation was observed. Stereological analysis at the suburethral area showed a significant difference in collagen presence in favor of CEC.

**Conclusions:**

The CEC implant showed adequate results when used as a suburethral sling with good integration to the host tissue, preserving its architecture.

## INTRODUCTION

Urinary incontinence affects millions of women in the World. It is estimated a prevalence ranging from 10 to 52% of the adult population (1).

Many surgical procedures have been proposed for the treatment of female SUI. Pubovaginal slings have shown good efficacy with satisfactory results in the long term follow-up (2). Autologous fascia has been widely used and became the treatment of choice for many women suffering from SUI (2, 3). The use of tension–free vaginal tape (TVT) started a new era of the use of heterologous materials which simplifies the procedure and decreases morbidity (4).

Several synthetic materials including polypropylene and polytetrafluorethylene (PTFE) have been used with this purpose despite the higher risk of infection and erosion (5, 6).

The cellulose exopolysaccharide has demonstrated effectiveness in different areas of surgery, acting as a conductor and inducing the healing process (7, 8).

In the present study we evaluated the interaction of the cellulose exopolysaccharide (CEC) with urethral tissue when used as a pubovaginal sling.

## MATERIALS AND METHODS

**Figure 1:**
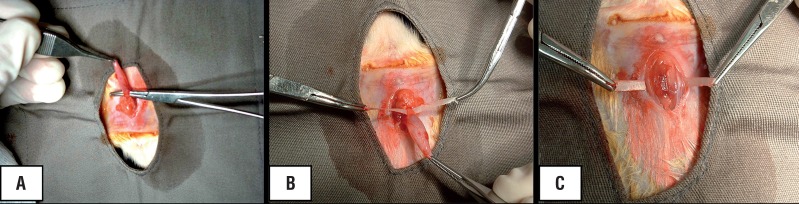
Surgical technique of implantation showing dissection of the urethra (A), positioning of the CEC at the suburethral space (B) and fixation to the abdominal wall (C).

Forty female Wistar rats with weight ranging from 215 to 297g were used in the present study. They were divided into 4 groups of 10. Two groups (A and B) were submitted to the implant of CEC that had 5cm in length and 0.3cm in width and groups C and D received the polypropylene mesh with the same dimensions. In both cases the surgical implantation occurred below the bladder neck with tension free fixation at the rectus muscle fascia (Figure-1).

Animals in subgroups A and C were sacrificed 4 weeks after the implant and animals in subgroups B and D after 12 weeks in order to evaluate tissue reaction and integration to the host. Bladder, urethra and abdominal musculature were removed as a single block keeping their anatomic relationship. The whole block was preserved in buffered 10% formalin for 15 days after which was processed for histological analysis.

Histological slides (5 microns) were processed using Haematoxilin-Eosin (HE) staining to evaluate inflammatory reaction. The following criteria were used for this purpose: (1) Absent: no inflammation or less than 5% at the analyzed area; (2) Slight: inflammatory reaction between 5 and 25%; (3) Moderate: Inflammatory reaction between 25 and 70%; (4) Intense: over 70% of the studied area. Collagen quantification was performed through stereological analysis and the slides were stained by Picrosirius red. Samples were analyzed by the same pathologist using 40X magnification.

Numeric variables were expressed by central tendency and dispersion. Kruskal-Wallis test was used to express continuous variables and Chi Square for categorical variables. The significance level to reject the nullity hypothesis was 5% (p<-0.05).

This research followed the rules of the experimental ethics code and the animal protecting laws practiced in Brazil. This study was approved by the Ethics Committee for animal study of the Institution.

## RESULTS

**Figure 2:**
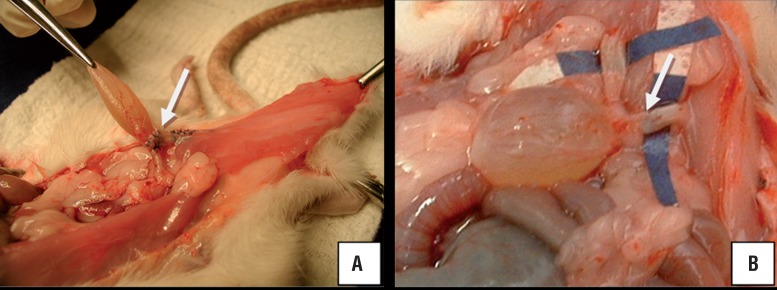
Demonstration of polypropylene (A) and CEC (B) in situ at 120 days sacrifice. Adequate incorcoporation to the implanted area.

No postoperative surgical complication was found in all animals. During sacrifice no atypical tissue reaction was observed as a consequence of the use of both materials. No fistula or abscess was found. Macroscopically, a good incorporation was observed (Figure-2).

Microscopically the subaponeurotic area showed intense inflammatory reaction in both groups with a significant presence of polymorphonuclear neutrophils in the analysis after 4 weeks. No atypical formation was observed.

**Figure 3:**
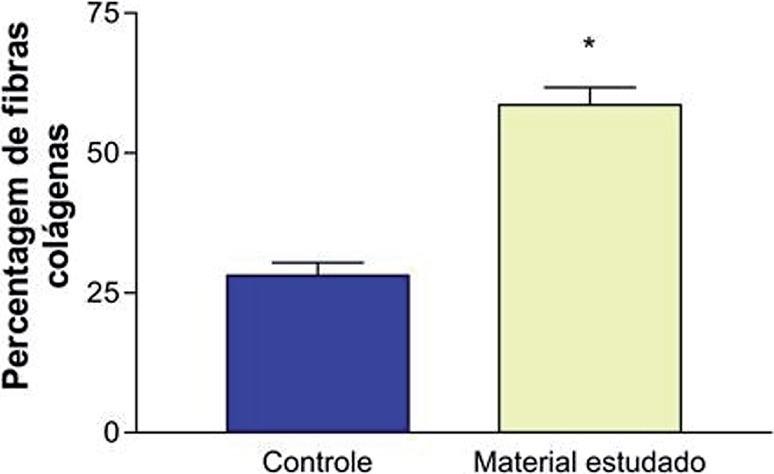
Analysis of collagen deposition in the suburethral space. Comparison between the biomaterials. *p<0.05.

The same analysis performed in animals sacrificed at 12 weeks showed a slight increase in collagen deposition at the subaponeurotic area in animals receiving CEC as compared to polypropylene mesh (25.90% vs 22.30), p=0.21 (Figure-3).

Stereological analysis at the suburethral area showed a significant difference of collagen presence in favor of CEC (58.60% vs 28.10), p=0.003.

**Figure 4:**
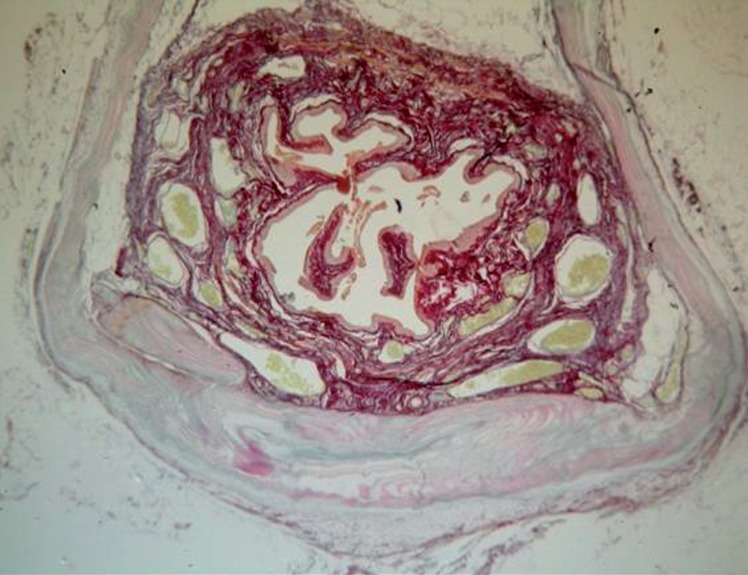
CEC sling in the suburethral space (red arrow) (HE, 40X).

The inflammatory reaction induced by CEC sling showed an intense proliferation of polymorphonuclear cells even in the late phase. Some angiogenesis with discrete neovascularizaton penetrating the new material was found. No capsule formation was found around the CEC implant (Figure-4).

The qualitative analysis concerning inflammation and the degree of necrosis in groups B and D in both suburethral and subaponeurotic regions showed similar results. The polypropylene group showed less inflammatory reaction than CEC in the suburethral area.

## DISCUSSION

The inflammatory reaction from a synthetic material in an animal model represents an important step in the evaluation of its future use in clinical trials. Several materials have been used as slings in the treatment of SUI and at the present polypropylene mesh is incorporated to various industrial mechanisms that are currently in clinical use (3, 5, 6, 9). The main reason to study a new material such as CEC is its physical properties and biocompatibility as well as the fact of being produced from a renewable material such as sugarcane sub product.

The cellulosic exopolysaccharide (CEC) has already been tested concerning cytotoxicity which has been evaluated through the adhesion index, nitric acid production and cellular viability of alveolar macrophages. These tests have been done in rats and no toxic reaction was found in the cell culture. There was also a high compatibility level in three cytotoxicity studies (10) which follow the recommendations of the Food and Drug Administration (FDA) that states that implantable medical devices and their constituents should not produce adverse reactions both locally and systemically, have no carcinogenic effect and produce no harm to the reproductive system and development (11).

CEC presents satisfactory physical properties such as elasticity, resistance to traction and flexibility allowing easy modulation in different shapes including the possibility of using as a sling. In an independent study in rats polypropylene mesh and CEC were implanted into the peritoneum and demonstrated that there was incorporation of both bio prosthesis without the presence of exudates and fistulas (12).

There is growing concern among surgeons using different types of industrial materials specially related to the risks of erosion, infection and biocompatibility status (5, 6, 13). In the early phase of the present study it was observed a high level of granulocytes infiltration as well as the presence of fibroblasts. These findings represent typical aspects of tissue repair involving the vascularization process. In this phase there is activation of the coagulation, platelets aggregation that involves its invasion by neutrophils, monocytes, macrophages and fibroblasts. This phenomenon is known as chemotaxis (13). The centripetal migration of endothelial cells in the area of the CEC implant interferes in the angiogenesis process justifying tissue remodeling and the integration of this new material in the suburethral area.

Microscopic analysis of the suburethral area showed a significantly elevated amount of collagen (30.5%) in the group that used CEC implant as compared to the control group. Erosion related to the use of the material was not found. A greater collagen deposition was observed in the suburethral region for both groups of materials when compared to subaponeurotic area.

This significant increase in collagen density is probably related to the interaction between cytokines and cells related to the healing process. Early research conducted to evaluate the biomechanics characteristics of CEC membrane demonstrated that after implantation there is an increase of its resistance to traction associated to the tissue integration. This phenomenon may represent a relevant point in favor of the increase of maintenance of continence rates when in clinical use (14).

In the present study a more intense inflammatory reaction was found at the suburethral area when compared to the control group in the 12 weeks group but this was not associated with urethral erosion.

Tissue reaction of polypropylene sling was studied in three different types of mesh which are different in relation to the structure and size of the pores (15). Other studies emphasize the role of these pores in allowing the filling with connective tissue, migration of immunocompetent cells and angiogenesis (16). Macroporous materials present greater molecular permeability and consequently allow quicker fibrinous fixation. They work as a biologic glue preventing the accumulation of secretions (2).

Histological analysis demonstrates a decrease in inflammatory reaction and fibrosis when a larger porous material is used as a sling. On the other hand lower porosity materials tend to facilitate capsule formation. This phenomenon did not occur with the CEC membrane.

More recently, small intestine submucosa (SIS) has been introduced as an option to be used as a sling in the treatment of SUI. This material is an acellular matrix that is produced from the intestinal submucosa of pigs. It is composed of collagen, growth factors, glycosaminoglycan and glycoprotein. The porosity is described as microscopic (17, 18).

Due to its composition SIS is infiltrated by the host cells that quickly proliferate and result in regeneration of local tissue in a well organized way. The authors concluded that there was complete absorption of the sling material due to the exceptional biocompatibility (18). Incontinence recurred in all patients probably due to the material absorption and loss of urethral support. Although no clinical study has proven this idea it is theoretically hypothesized that materials to be used with this purpose must stay in situ for a minimum of 12 weeks.

CEC was not affected by the degradation process and no acute complications such as abscess formation or extrusion or fistulas related to the implantation of this material. We believe that the presence of collagen coating the suburethral mucosa may be responsible for the preservation of this tissue architecture and viscoelastic characteristics (12, 14). This suburethral support probably reduces the risks of erosion which is one of the main complications associated with the use of synthetic slings.

It is important to emphasize that the objective of the present research was not to evaluate the functional properties of the CEC implant since no tension was applied over it. The absence of suburethral erosion and reduced inflammatory reaction as well as the tendency for incorporation in the long term as demonstrated by the angiogenesis process may be the most important features to be taken in account.

## CONCLUSIONS

The CEC implant was shown to be appropriate when used as a suburethral sling in rats. Adequate integration to the host tissue and preservation of its architecture were the main findings of the study. The utilization of CEC may be considered as an ideal material to be used as a sling but the impact of its use in clinical practice still has to be tested.
